# Supplementary material for: New Insights about CuO Nanoparticles from Inelastic Neutron Scattering
Source: Nanomaterials (Basel). 2019 Feb 26;9(3):312. doi: 10.3390/nano9030312 (PMC6473763; doi:10.3390/nano9030312)

Electronic Supplementary Information (ESM)

New Insights about CuO Nanoparticles from Inelastic Neutron Scattering

Elinor C. Spencer[1], Alexander I. Kolesnikov[2], Brian F. Woodfield[3] and Nancy L. Ross[1,*]

[1] Dept. of Geosciences, Virginia Polytechnic Institute and State University, Blacksburg, Virginia 24061, U.S.A.
[2] Neutron Scattering Division, Oak Ridge National Laboratory, Oak Ridge, Tennessee 37831, U.S.A
[3] Dept. of Chemistry and Biochemistry, Brigham Young University, Provo, Utah 84602, U.S.A.

INFORMATION ABOUT ELECTRONIC SUPPLEMENTARY MATERIAL. The ESM includes additional data and figures of the spin-wave excitations of 15 nm CuO and particle-size dependence of CuO INS spectra.

Figure S1. S(Q,E)·E/[n(E,T)+1] intensity of the spin-wave excitations of 15 nm CuO particles at different temperatures obtained from the S(Q,E) spectra measured with E_i=25 meV and summed over energy range E=3 to 10 meV.

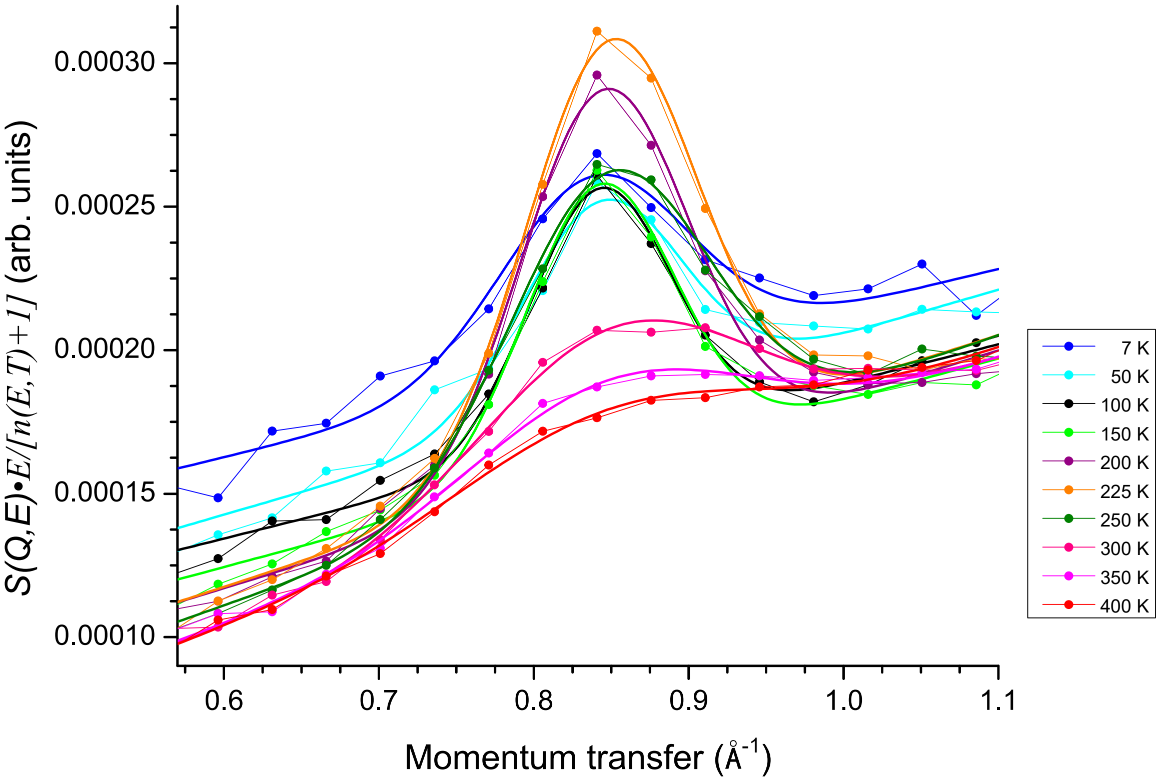

Figure S2. S(Q,E)·E/[n(E,T)+1] intensity of the spin-wave excitations of 15 nm CuO particles at different temperatures obtained from the S(Q,E) spectra measured with E_i=50 meV and summed over energy range *E*=6 to 16 meV.

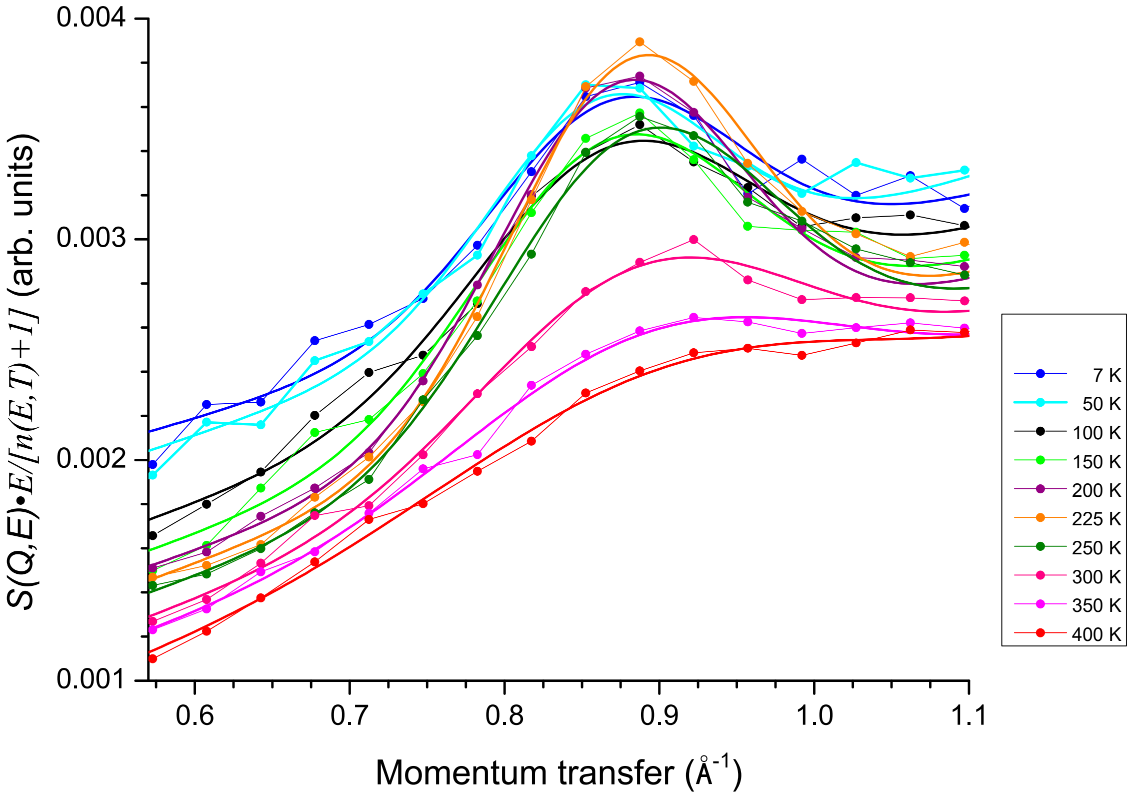

Figure S3. Temperature dependence of the spin-wave excitation intensity of 15 nm CuO particles obtained from the plots in Figs. S3 and S4 as integral intensities of the Gaussian peaks above the sloped background.

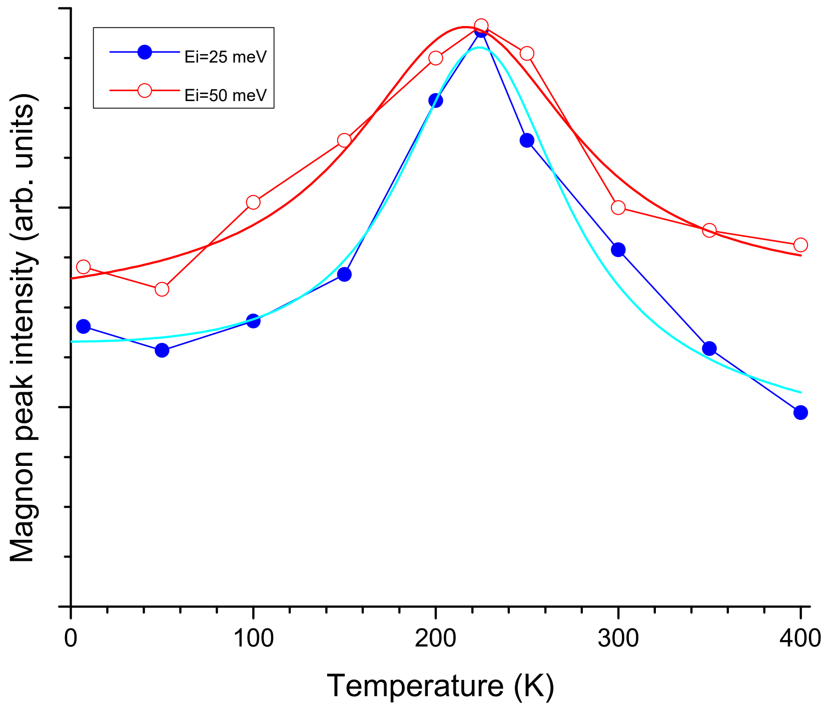

Figure S4. INS spectra for 8 and 25 nm CuO nanoparticles. Both spectra were collected with E_i = 50 meV and at T = 7 K. The spectra are plotted with the same intensity scale (right).

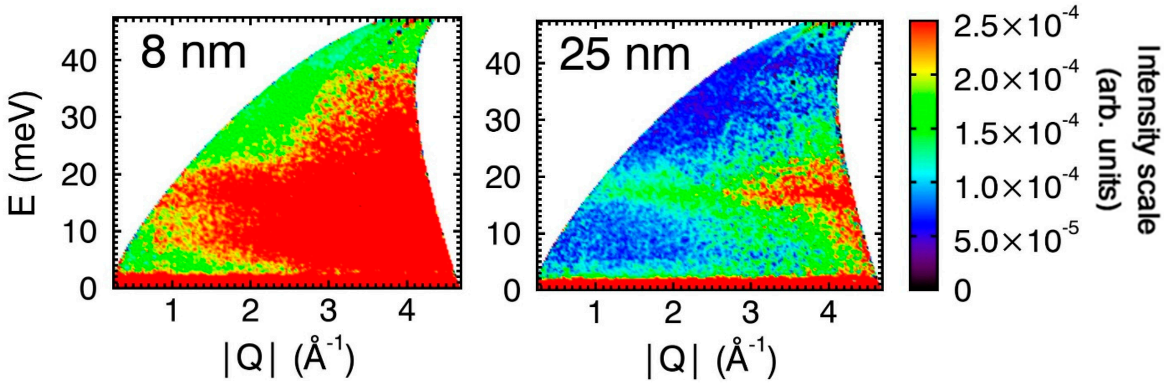

Supplement: Supplementary file 1 [file nanomaterials-09-00312-s001.pdf]
